# Supplementary material for: Multi-omics profiling of collagen-induced arthritis mouse model reveals early metabolic dysregulation via SIRT1 axis
Source: Sci Rep. 2022 Jul 12;12:11830. doi: 10.1038/s41598-022-16005-9 (PMC9276706; doi:10.1038/s41598-022-16005-9)
Supplement: Supplementary file 1 — Supplementary Information. [file 41598_2022_16005_MOESM1_ESM.pdf]

Supplementary Table 1. Sample sizes for multi-omics experiments on CIA mice.

Timepoint	Treatment	Paw_RNAseq	Plasma_Metabolomics	Paw_MALDI-MS
2 days	CIA	18	18	NA
2 days	Ctrl	6	6	NA
2 weeks	CIA	15	17	6
2 weeks	Ctrl	6	6	5
3 weeks	CIA	14	18	NA
3 weeks	Ctrl	6	6	NA
4 weeks	CIA	16	17	6
4 weeks	Ctrl	5	6	5
6 weeks	CIA	17	18	6
6 weeks	Ctrl	5	6	5
7 weeks	CIA	17	17	NA
7 weeks	Ctrl	5	5	NA
8 weeks	CIA	16	16	NA
8 weeks	Ctrl	5	5	NA
10 weeks	CIA	15	16	6
10 weeks	Ctrl	5	5	5

Supplementary Table 2. Top 20 activated and top 20 inhibited upstream regulators from IPA on human synovium RNA-seq data.

One exception here is that SIRT1 was not among the top 20 inhibited upstream regulators when considering its z score, but it was still significant at the stage of early RA.

Upstream Regulators	Molecule Type	zScore. Arthralgia	BH_pval.Arthralgia	zScore. UA	BH_pval. UA	zScore. Early RA	BH_pval. Early RA
**lipopolysaccharide**	chemical drug	4.95	0.014537887	8.998	4.11E-07	9.43	5.49E-14
**IL1B**	cytokine	4.907	0.021379927	7.602	2.52E-07	7.375	4.66E-10
**TNF**	cytokine	2.958	0.001881826	7.167	4.63E-08	7.269	7.53E-11
**IFNG**	cytokine	3.562	7.87E-05	5.294	1.98E-07	6.855	5.62E-13
**poly rI:rC-RNA**	biologic drug	NA	1	6.346	0.001285	6.85	1.47E-05
**CSF2**	cytokine	2.991	0.038963384	5.762	2.34E-05	6.22	4.30E-12
**Interferon alpha**	group	3.072	0.095024112	4.657	0.001586	6.22	1.21E-09
**IL6**	cytokine	3.391	0.015684944	6.085	6.48E-07	5.708	7.73E-16
**OSM**	cytokine	3.287	0.149468491	5.45	0.004161	4.29	0.005606
**TLR9**	transmembrane receptor	NA	1	4.996	0.008627	5.441	4.76E-07
**PTPRR**	phosphatase	5.379	7.87E-05	4.264	0.00075	3.162	0.194891
**IL33**	cytokine	4.014	0.01118701	4.799	3.81E-06	5.364	8.78E-10
**MYD88**	other	NA	1	5.35	0.001759	5.123	0.000104
**CD28**	transmembrane receptor	2.21	0.149194061	4.904	0.032463	5.301	3.41E-09
**RELA**	transcription regulator	3.682	0.028405697	5.019	1.58E-05	5.288	6.19E-09
**NFkB (complex)**	complex	2.115	0.000412795	4.429	1.40E-06	5.241	1.58E-12
**IL2**	cytokine	2.759	0.039192814	4.569	0.000191	5.225	4.14E-16
**TNFSF12**	cytokine	4.909	0.000165264	5.183	2.87E-06	4.81	2.50E-07
**AHR**	ligand-dependent nuclear receptor	3.531	0.055169976	3.275	0.000616	5.024	1.20E-09
**TLR7**	transmembrane receptor	NA	1	4.561	0.032646	5.018	3.37E-06
**CITED2**	transcription regulator	NA	1	-5.331	0.018805	-5.151	0.000432
**Firre**	other	-4.914	1.24E-05	-4.69	6.73E-05	-3.742	0.006452
**Tgf beta**	group	-4.659	0.000107735	-1.974	2.42E-06	-2.674	1.01E-09
**PPARG**	ligand-dependent nuclear receptor	-2.987	0.464494386	-4.648	0.037067	-2.719	0.01186
**ZFP36**	transcription regulator	-4.258	0.017980268	-4.383	1.98E-07	-4.605	6.57E-08
**NUPR1**	transcription regulator	-1.921	0.394578913	-2.06	0.235364	-4.371	0.000475
**TP53**	transcription regulator	-4.26	0.304135769	-3.912	0.067025	-3.736	0.0039
**EHMT1**	transcription regulator	-4.082	0.007006118	-3.578	0.005132	-1.678	0.009123
**Irgm1**	other	NA	1	-3.063	0.030323	-4.022	8.88E-05
**IL1RN**	cytokine	-3.201	0.026427214	-3.626	0.000645	-4.018	8.91E-05

**SP1**	transcription regulator	-3.953	0.017617377	-1.95	0.00036	0.311	0.002874
**SS18**	transcription regulator	-3.742	0.001326942	-2.111	0.003711	-1.508	0.000292
**CTLA4**	transmembrane receptor	-2.797	0.038563448	-3.089	0.001759	-3.659	4.74E-09
**DAP3**	other	-3.317	2.52E-06	-3.317	6.55E-08	-3.606	1.58E-13
**LONP1**	peptidase	-2.887	0.334364038	-1.678	0.027521	-3.606	0.009718
**ESRRA**	transcription regulator	-3.604	0.031263041	NA	1	-0.077	0.13618
**CDKN1A**	kinase	NA	1	-3.054	0.030854	-3.509	5.81E-06
**LILRB4**	other	-3.211	0.022828467	-3.211	0.00304	-3.497	8.08E-06
**NORAD**	other	-3.45	0.021379927	-1.706	0.045173	-2.121	0.02357
**DUSP1**	phosphatase	-3.151	0.040947334	-3.435	2.87E-06	-3.441	6.00E-06
**SIRT1**	transcription regulator	NA	1	NA	1	-1.36	0.043221

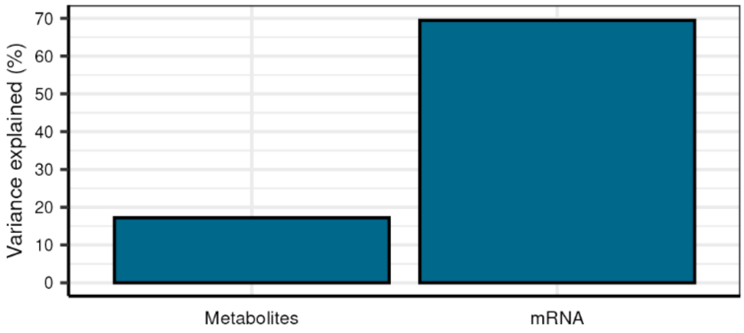

Fig. S1: Cumulative variance explained by all factors in each data modality.

MOFA identified in total 6 factors, based on the criterion that a factor should account for > 2% of the variance in at least one omics dataset. All the factors together explained approximately 20% of variance in metabolomics dataset, and approximately 70% of variance in transcriptomics dataset.

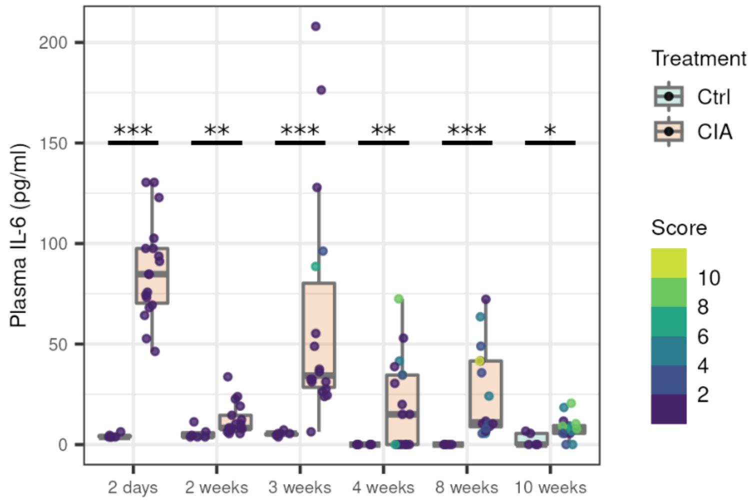

Fig. S2: Plasma concentration of IL-6.

The cytokines and chemokines were measured by mouse magnetic Luminex assays. The dots on the boxplots represent individual mouse samples. At each time point, the sample size for controls is ⩾5 and the sample size for CIA is ⩾ 16. The color of each dot represents the arthritis score of the mouse (sum of 4 paws). Two-tail Welch t tests were performed ($p < 0.05$ *, $p < 0.01$ **, $p < 0.001$ ***).

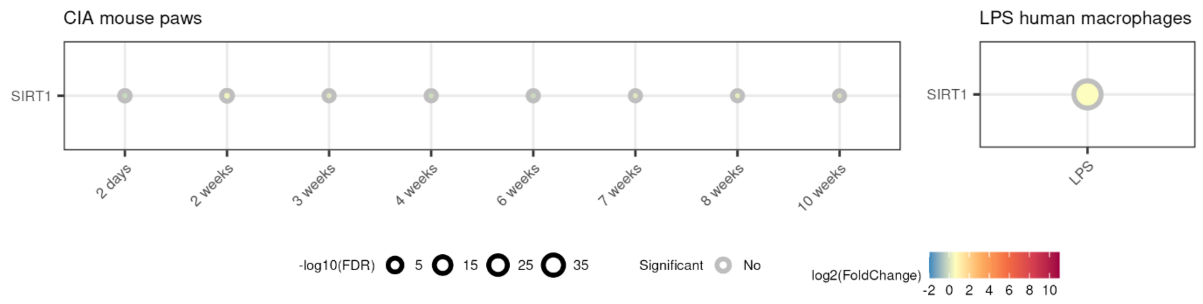

Fig. S3: Differential gene expression of SIRT1.

Differential gene expression of SIRT1 in the RNA-seq studies of CIA mouse paws and LPS-stimulated human macrophages. Significance was decided by cutoffs of FDR < 0.05 and fold change > 1.5 in up or down direction. Legend scale is the same as in Fig. 8a-c.

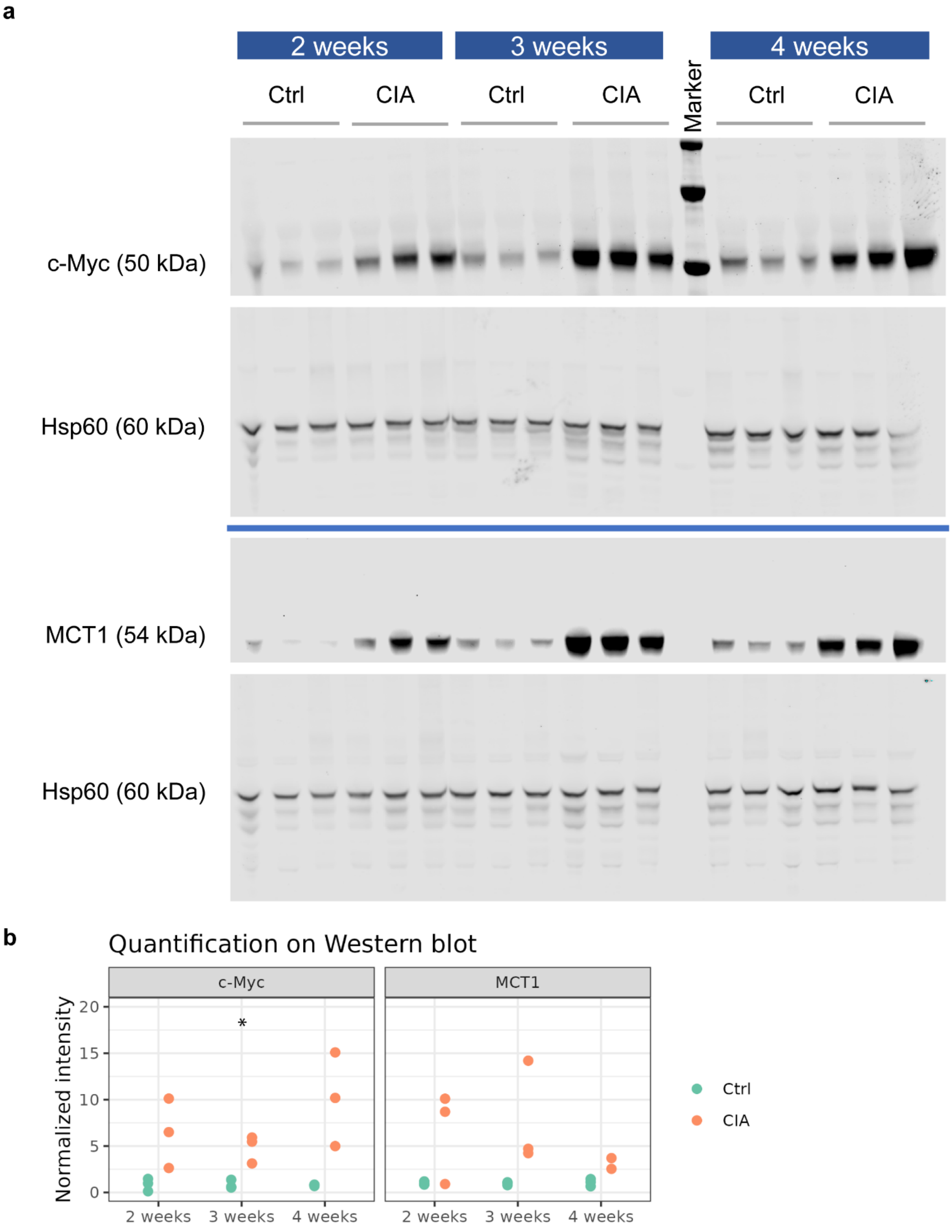

Fig. S4: Western blot on glycolysis-related proteins c-Myc and MCT1.

a Representative Western blot images for c-Myc and MCT1. Hsp60 was used as a loading control. **b** Normalized intensity for c-Myc and MCT1 (mean of 2 independent Western blots). The signal intensity was first normalized with the Hsp60 intensity, and then normalized to the mean values of the control samples at each respective time point. On the figure, each dot represents one mouse sample. Quantification for MCT1 at 4 weeks has one missing value, due to signal overload. Two-tail Welch t tests were

performed ($p < 0.05$ *, $p < 0.01$ **, $p < 0.001$ ***) between CIA and control samples at each respective time point.

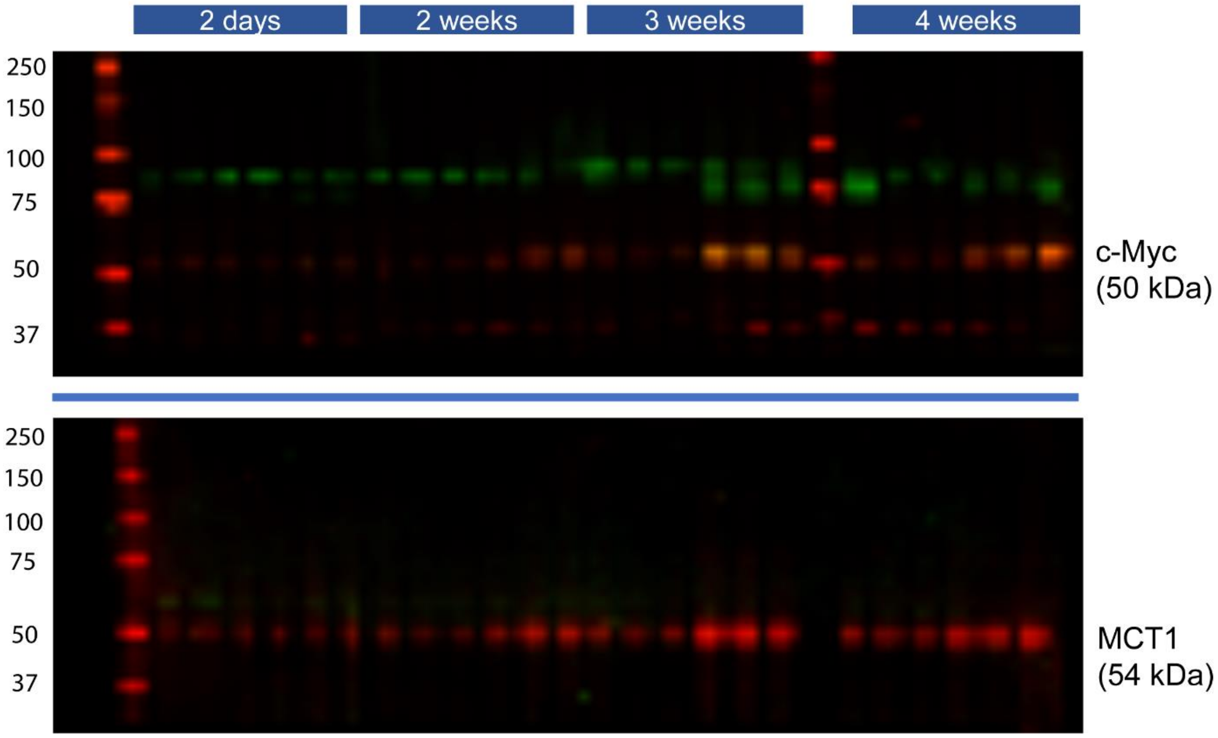

Fig. S5: Preview image for Western blot in Fig. S4.

Detection of c-Myc was achieved by c-Myc Mouse mAb and IRDye® 680RD Goat anti-Mouse IgG secondary antibody. The green bands show signals from another protein target (different host) that is not relevant for our study. Detection of MCT1 was achieved by MCT1 Mouse polyclonal and IRDye® 680RD Goat anti-Mouse IgG secondary antibody. For preview on the Odyssey infrared imager (LI-COR), preliminary and low-resolution image was captured in the defined area of the blot, before adjusting the scan area and re-scanning for a high-resolution image. The blots were physically cropped at 25 kDa before incubation with c-Myc and MCT1 antibodies.
